# Supplementary material for: Mapping the last mile: Micro-stratification for sustained visceral leishmaniasis elimination in Bangladesh
Source: PLoS Negl Trop Dis. 2026 May 11;20(5):e0013504. doi: 10.1371/journal.pntd.0013504 (PMC13175496; doi:10.1371/journal.pntd.0013504)
Supplement: S1 Table — (PDF) [file pntd.0013504.s001.pdf]

Division	Endemic Mouza (%) per division	Endemic Districts	Case Reporting Upazila	No. of Endemic Mouza (%)	Endemicity		
					Low	Moderate	High
Mymensingh	35.28%	Jamalpur	6	34 (7.94%)	31	2	1
		Mymensingh	9	115 (26.87%)	81	21	13
		Sherpur	2	2 (0.47%)	2	0	0
Dhaka	28.74%	Dhaka	3	5 (1.17%)	5	0	0
		Gazipur	5	22 (5.14%)	18	2	2
		Kishoreganj	3	5 (1.17%)	4	0	1
		Manikganj	3	9 (2.1%)	9	0	0
		Munshiganj	2	2 (0.47%)	2	0	0
		Narayanganj	1	2 (0.47%)	2	0	0
		Narsingdi	3	8 (1.87%)	7	1	0
		Rajbari	1	2 (0.47%)	1	1	0
		Shariatpur	2	2 (0.47%)	0	1	1
		Tangail	12	66 (15.42%)	58	6	2
Rajshahi	21.50%	Bogura	3	4 (0.93%)	4	0	0
		Chapai Nawabganj	1	4 (0.93%)	4	0	0
		Naogaon	7	10 (2.33%)	10	0	0
		Natore	3	7 (1.64%)	6	0	1

		Pabna	7	40 (9.35%)	34	4	2
		Rajshahi	5	9 (2.1%)	9	0	0
		Sirajganj	5	18 (4.21%)	15	2	1
Khulna	5.61%	Bagerhat	1	1 (0.23%)	1	0	0
		Jhenaidah	3	4 (0.93%)	4	0	0
		Khulna	1	4 (0.93%)	3	0	1
		Kushtia	2	8 (1.87%)	6	0	2
		Magura	1	2 (0.47%)	2	0	0
		Meherpur	2	3 (0.7%)	3	0	0
		Narail	1	2 (0.47%)	2	0	0
Rangpur	3.74%	Dinajpur	7	11 (2.57%)	9	1	1
		Kurigram	2	2 (0.47%)	2	0	0
		Rangpur	1	3 (0.7%)	2	0	1
Barishal	3.04%	Bhola	1	1 (0.23%)	1	0	0
		Patuakhali	4	11 (2.57%)	9	2	0
		Pirojpur	1	1 (0.23%)	1	0	0
Chattogram	1.64%	Brahmanbaria	1	1 (0.23%)	1	0	0
		Chandpur	1	1 (0.23%)	1	0	0
		Chattogram	1	1 (0.23%)	1	0	0
		Cumilla	1	4 (0.93%)	4	0	0
Sylhet	0.47%	Sunamganj	4	1 (0.23%)	1	0	0

		Sylhet	1	1 (0.23%)	1	0	0
**Total**	**100%**		**119**	**428 (100%)**	**356**	**43**	**29**
